# At-Risk Elementary School Children with One Year of Classroom Music Instruction Are Better at Keeping a Beat

**DOI:** 10.1371/journal.pone.0077250

**Published:** 2013-10-10

**Authors:** Jessica Slater, Adam Tierney, Nina Kraus

**Affiliations:** 1 Auditory Neuroscience Laboratory, Northwestern University, Evanston, Illinois, United States of America; 2 Department of Communication Sciences, Northwestern University, Evanston, Illinois, United States of America; 3 Institute for Neuroscience, Northwestern University, Chicago, Illinois, United States of America; 4 Department of Neurobiology and Physiology, Northwestern University, Evanston, Illinois, United States of America; 5 Department of Otolaryngology, Northwestern University, Chicago, Illinois, United States of America; UNLV, United States of America

## Abstract

Temporal processing underlies both music and language skills. There is increasing evidence that rhythm abilities track with reading performance and that language disorders such as dyslexia are associated with poor rhythm abilities. However, little is known about how basic time-keeping skills can be shaped by musical training, particularly during critical literacy development years. This study was carried out in collaboration with Harmony Project, a non-profit organization providing free music education to children in the gang reduction zones of Los Angeles. Our findings reveal that elementary school children with just one year of classroom music instruction perform more accurately in a basic finger-tapping task than their untrained peers, providing important evidence that fundamental time-keeping skills may be strengthened by short-term music training. This sets the stage for further examination of how music programs may be used to support the development of basic skills underlying learning and literacy, particularly in at-risk populations which may benefit the most.

## Introduction

The ability to keep a beat lies at the heart of music-making. A typically-developing child can maintain a steady beat as young as four years old[1], and even infants can “feel the beat”, demonstrating sensitivity to metrical structure well before they are able to synchronize to it themselves[2,3]. Though the emergence and mastery of rhythm skills can continue through to adulthood, rhythmic competence can depend greatly on the extent of engagement with music [4-7]. While a child who struggles to speak his or her native language may be assessed for a language deficit, individuals who fail to develop basic musical skills (even those engaged in musical training) are often simply considered “unmusical”. However, performance on simple tapping tasks relates with a variety of critical abilities outside the musical domain, from cognitive and linguistic skills[8,9] to handwriting[10]. Moreover, abnormal rhythmic performance is associated with language deficits such as dyslexia[11-13] as well as attention-related deficits[14]. The potential for music classes to strengthen fundamental temporal processing mechanisms therefore holds great promise for educators and clinicians. 

Despite significant advances in the understanding of sensorimotor synchronization skills across the lifespan, including the effects of musical expertise[15,16], no studies to our knowledge have investigated the relationship between classroom music instruction and beat-keeping abilities in young children, at an age when both rhythm and literacy skills are emerging. While the ability to attend to rhythmic patterns and maintain a steady beat increases between the ages of 5 and 7 [1,5], it has been suggested that rhythm abilities reach adultlike “nonmusician” levels by ages 7 to 8, and may not develop further without additional training [5]. Given the evidence for links between rhythm skills and reading ability, it is of great interest to educators and clinicians to determine whether musical training can support the development of reading skills by strengthening underlying temporal processing mechanisms.

The present study was carried out in collaboration with Harmony Project, a non-profit organization providing free music education to underserved children in gang-reduction zones of Los Angeles. We used continuation and paced tapping paradigms to assess beat-keeping skills in a group of elementary school children who had received one year of musical training, compared with a matched group of untrained controls. We hypothesized that musical training strengthens basic timekeeping skills, resulting in greater accuracy in keeping a beat in the musically-trained children. 

## Methods

### Ethics Statement

All experimental procedures and forms were approved by the Northwestern University Institutional Review Board.

### Participants

Participants were recruited from the waitlist of Harmony Project (http://www.harmony-project.org). Researchers from Northwestern University traveled to Los Angeles, CA, for approximately three weeks in the summer of 2011 to collect data. Following the initial assessment, participants were randomly assigned by the Northwestern research group to training and control groups. Then, minor adjustments were made to ensure the groups were matched for sex, handedness and non-verbal I.Q., assessed using the non-verbal subtest of the Wechsler Abbreviated Scale of Intelligence[17] (see Table 1). All subjects had normal hearing and no history of learning or neurological disorders based on parental report.

**Table 1:** Group comparison pre-training.

	**Number of children**		**Total**	**Statistical comparison**
**Sex**	**Control group**	**Training group**		
Male	12	12	24	
Female	19	17	36	χ^2^=0.044, p= 0.521
			60	
**Age**	**Control group**	**Training group**		
6 years	2	2	4	
7 years	9	6	15	
8 years	14	16	30	
9 years	6	5	11	
			60	
**Mean age**	**8.2 years**	**8.3 years**		F(1,58)=0.137, p=0.713
**Non-verbal IQ**				
Mean score (SD), WASI non-verbal IQ	52.32 (10.4)	52.55 (10.0)		F(1,58)=0.008, p=0.931

The training group (n=29, 17 females) began music classes with the Harmony Project in August of 2011, and the control group (n=31, 19 females) began music classes the following summer. Given the high demand for the program, this still constituted a reduction in total wait time for the control group children and therefore was considered ethically acceptable for the purposes of the study. 

None of the children had previously received musical training of any kind. This was confirmed at the initial assessment and again when the participants returned for testing 12 months later. Importantly, the subject selection process meant that all participating families wanted their children to be in the music program; as a result it can be assumed that the groups were as well-matched as possible in terms of student motivation and parental support. Over the 12 month period, Harmony Project and Northwestern staff invested significant efforts to maintain contact with control participant families via mail, phone and community events to ensure continued engagement in the project. 

Informed written consent was obtained from caretakers or guardians on behalf of the children participating in the study in their language of preference (either English or Spanish) and informed written assent was obtained in English from the participants. Participants were monetarily compensated for their time.

### Musical training

The musical training was delivered in the context of an existing community-based program: the children in the training group attended one out of four music programs offered by Harmony Project across the Los Angeles area. They followed the standard Harmony Project curriculum which begins with two, one-hour musicianship classes per week. The musicianship curriculum targets several key learning outcomes, including basic rhythm and pitch skills, performance, improvisation and composition, musical awareness and musical terms, basic recorder performance, and knowledge of the instruments in an orchestra (for a summary of the musicianship learning objectives, see Table 2). 

**Table 2:** Musicianship learning objectives.

**Rhythm**
Identify, read and perform basic note/rest values, measures, bar lines, meters (e.g. music math)
Identify and perform simple rhythmic patterns and ostinatos with a steady beat
**Pitch**
Name lines/spaces on the staff (treble and bass clef, basic understating of grand staff and C clef)
Identify and perform simple melodic patterns
Match and adjust pitch, follow pitch direction through movement
Sing and identify major and minor scales
**Performance**
Sing and perform independently and in groups, on pitch and in rhythm, blending timbres
Follow a conductor for dynamics, tempo, and cues
Exhibit appropriate rehearsal etiquette
Students echo short rhythms and melodic patterns (call and response)
**Improvisation and Composition**
Improvise "answers" in the same style to given rhythmic and melodic phrases (call and response)
Improvise simple rhythmic variations and melodic embellishments on familiar melodies
Create and arrange short songs and instrumental pieces within specified guidelines
Write and perform simple compositions
**Musical Awareness**
Explain personal preferences for music and styles using appropriate terminology for music, music notation, music instruments and voices
Identify instruments and their sounds, including instruments from various cultures
Listen to music, analyze and describe structure/emotion
**Musical Terms**
Melody, rhythm, and harmony
Beat, measure, bar line, repeat sign, double bar
Tempo (lento, adagio, allegro & presto)
Dynamics (p, mp, mf, f, crescendo and decrescendo)
Whole, half, dotted half, quarter, and eighth notes, whole, half, quarter rests
Time signature (4/4, 2/4, 3/4)
Verse, chorus, tutti, solo, duet
Scale, chord, sharp, flat, key signature, intonation
Grand Staff, treble clef, bass clef, C clef
**Orchestra Instrumentation**
Conductor
Strings: Violin, Viola, Cello, Double Bass, Guitar, Harp
Winds: Recorder, Flute, Clarinet, Oboe, Bassoon, Saxophone
Brass: French horn, Trumpet, Trombone, Tuba
Percussion: Timpani, Cymbals, Snare Drum, Drum Set, etc.
Piano and Keyboard

Once students attained a level of basic competence in musicianship (as assessed by their classroom teacher) they began instrumental lessons. Twenty students began instrumental lessons in January of 2012; the remaining nine students continued in musicianship class until the end of the school year, at which point they passed the musicianship assessment and began instrumental training the following fall. These nine children had therefore not begun instrumental classes at the time of our second assessment. The children chose their instrument from currently available options, which are dependent on teacher and instrument availability (details of the instruments played by the participants as well as the programs attended are included in Table 3). Since Harmony Project provides music instruction outside the public school system, teachers are primarily professional musicians and private instructors (further details about teaching staff and Harmony Project programs can be found at http://www.harmony-project.org/).

**Table 3:** Instrumental training summary.

**Harmony Project Program**	**Typical class participation**		**Number of children**
Alexandria Elementary School	One-hour instrumental classes twice a week plus a two hour string ensemble rehearsal each week		4
Beyond the Bell	Twice-weekly two-hour ensemble rehearsals. These include pull-out sectional rehearsals, which are similar to large instrumental classes at other sites.		14
EXPO Center (YOLA)	One-hour instrumental music classes each week and a three hour ensemble rehearsal each week.		6
Hollywood	One-hour instrumental classes twice a week plus a three-hour ensemble rehearsal (concert band) each week.		5
	TOTAL		**29**
**Instruments (started Jan 2012)**			
Bass			4
Cello			3
Flute			1
French horn			1
Trumpet			10
Viola			1
Musicianship/recorder for full year		*These students continued in musicianship classes until the end of the year and began instruments in the fall of 2012*	9
		TOTAL	**29**

After one year, in summer of 2012, all participants were assessed by a research group from Northwestern University.

The tapping measures reported in this study were incorporated into the second year of an ongoing longitudinal study assessing the transfer effects of musical training to non-musical outcomes, such as auditory skills and language development. Tapping paradigms have been adopted in our lab as part of an emerging line of rhythm-related research, and we were able to add these measures to the existing protocol to compare tapping abilities between a musically-trained group and a matched control group. 

Therefore, the tapping measures were *not* part of the initial assessment carried out prior to training. The tapping assessment was added to an existing 4-5 hour testing protocol which included neural, perceptual and cognitive assessments: following an initial audiometric screening, the order of the remaining assessments was determined primarily by availability of testing space and was blind to group assignment. Therefore, while general fatigue could play a role in tapping performance, there should have been no bias between testing groups as far as whether the tapping assessment was carried out near the beginning or the end of the testing session. None of the other tests involved repetitive or rhythmic motor tasks which would have resulted in motor fatigue, nor did they include any other music-related tasks which might have primed participants to perform in any particular way. The tapping assessments themselves took approximately 15 minutes.

### Tapping Tests

The tapping tests were administered one-on-one with an experimenter. The apparatus used was a NanoPad2 (KORG, Japan). It is a small rectangular plastic device containing 16 square rubber pads, each approximately two inches across. During the training phase, the experimenter familiarized the participant with the device, verbally instructing them to pick one of the pads and tap on it as if they were hitting a drum. In this training phase, tapping on the pad resulted in a drum sound; the experimenter demonstrated this by tapping once or twice on the pad. If the subject did not tap with sufficient force, no drum sound was generated and the experimenter provided further coaching to the subject to hit the pad more forcefully (e.g. “remember to hit the pad as if you are hitting a drum, rather than pressing a button”). After the subject had demonstrated 10-15 successful taps, the experimenter went on to begin the first tapping test. Tap times were recorded by custom-written software in Python. The sequence of conditions was controlled by a computer program developed in Python, such that the two paced conditions were administered first, beginning with the slowest tempo, then the continuation conditions also beginning with the slowest tempo. 

### Continuation Tapping Test

The experimenter explained that the subject would hear a drum sound presented at a regular beat, and that the subject should tap along to the beat such that the subject’s tap occurred at the exact same time as the sound that they heard. They were instructed to continue tapping at the same rate after the sound presentations stopped, until the experimenter asked them to stop tapping. The time period during which the subject continued to tap was equal to the duration of the pacing period. To give the subject ample time to synchronize to the beat at the beginning of each condition, the drum sound was presented twenty times before it was removed. Two different tempi were presented, 1.5 and 2 Hz. (These rates were chosen because they overlap with the rate of stressed syllable production in normal conversational speech[18].) The continuation tapping task lasted approximately five minutes.

### Paced tapping test

Subjects were asked to tap along with an isochronous auditory pacing stimulus for twenty beats before data collection began. In this condition, however, the pacing stimulus continued throughout the entire test (for another twenty beats), and participants were simply instructed to tap along to the beat and stop tapping once the beat stopped. Data was only recorded during this second half of the test (i.e. the last twenty beats). Two different tempi were presented, 1.5 and 2 Hz. The paced tapping task lasted approximately five minutes.

### Analysis

All tapping test analyses were conducted using custom-written software in Python. Both variability and accuracy of the tap times were calculated using methods based on previous work investigating individual differences in tapping ability [15]. Only taps produced after the first twenty pacing beats were used in the analyses. First, to remove instances where the subject did not tap with enough force to elicit a response from the tapping pad, inter-onset intervals (IOIs) exceeding 1.7 times the target IOI were removed from analysis. Further, cases in which tapping on the pad resulted in a double-tap (two taps very close together in time, possibly because of a small bounce occurring upon contact with the tapping pad) were corrected by removing the second of the two taps, with the assumption that the first tap was the tap the subject intended to produce. This was only done when the IOI between the two taps was a small fraction of the target IOI (less than 100 ms). After these cases were removed, the mean interval between each pair of taps was calculated and then compared with the target periodicity, with a smaller mean error indicating greater accuracy in maintaining the beat once the pacing stimuli had stopped. 

This mean error was calculated in two different ways: first, the absolute value of the difference between the target IOI and the tapping IOI was calculated, giving a measure of unsigned error, i.e. a measure of the extent to which the tapping IOI followed the target IOI, regardless of whether the subject tapped too quickly or too slowly. Second, the signed difference between the target IOI and the tapping IOI was calculated, giving a measure of signed error; a positive signed error indicates that the subject tapped too quickly relative to the target IOI, while a negative signed error indicates that the subject tapped too slowly. Both measures of error were divided by the target IOI so that error was expressed as a fraction of the target tempo. Composite accuracy measures were also calculated by averaging across both conditions[9]. Tapping variability was assessed by calculating the standard deviation of the IOIs, then dividing the result by the target IOI to calculate the coefficient of variation. Finally, to investigate whether drift was occurring (i.e. whether subjects were gradually speeding up or slowing down), we calculated the lag-1 autocorrelation of the sequence of IOIs. (If, as described above, IOIs were removed from analysis as a result of missing taps, those IOIs forming uninterrupted sequences were analyzed separately and averaged to form a mean lag-1 autocorrelation.) Positive autocorrelations indicate that drift is occurring, while negative autocorrelations indicate the presence of error correction (i.e. the subject is returning to the target IOI whenever they happen to deviate from it).

## Results

A series of repeated measures ANOVAs were run on each continuation and paced tapping measure to determine whether the rhythmic abilities of the musically-trained children differed from those of the control children. Tempo (2 Hz versus 1.5 Hz) was included as the within-subjects variable and group (musically trained versus control) was included as the between-subjects variable. For unsigned accuracy in the continuation condition there was a main effect of group (F(1,56) = 6.45, p = 0.0139): musicians performed more accurately (median composite error = 3.14%) compared to nonmusicians (median composite error = 4.81%). (Shapiro-Wilk’s test for non-normality revealed that all data points except for lag 1 autocorrelation were non-normally distributed at p < 0.05; as a result we report medians and use Wilcoxon rank sum tests rather than t-tests for post-hoc analyses.) (Nonmusician performance, at 4.81% composite error, was less than the 10.6% error reported for nine-year-old typically developing children by Corriveau and Goswami [11]. The worse performance reported by Corriveau and Goswami may be due to the fact that they used a longer continuation interval, giving subjects more time to drift away from the beat.) There was also a main effect of group for continuation signed accuracy (F(1,56) = 7.08, p = 0.0102): musicians sped up less relative to the target tempo (median composite error = 1.74%) compared with nonmusicians (median composite error = 4.81%). There were no main effects for paced unsigned accuracy, paced signed accuracy, continuation variability, paced variability, paced lag 1 autocorrelation, and continuation lag 1 autocorrelation. There was an interaction between group and condition for paced variability (F(1,56) = 5.60, p = 0.0214), but post-hoc Wilcoxon rank sum tests revealed no significant group differences (p > 0.05). 

**Figure 1:**
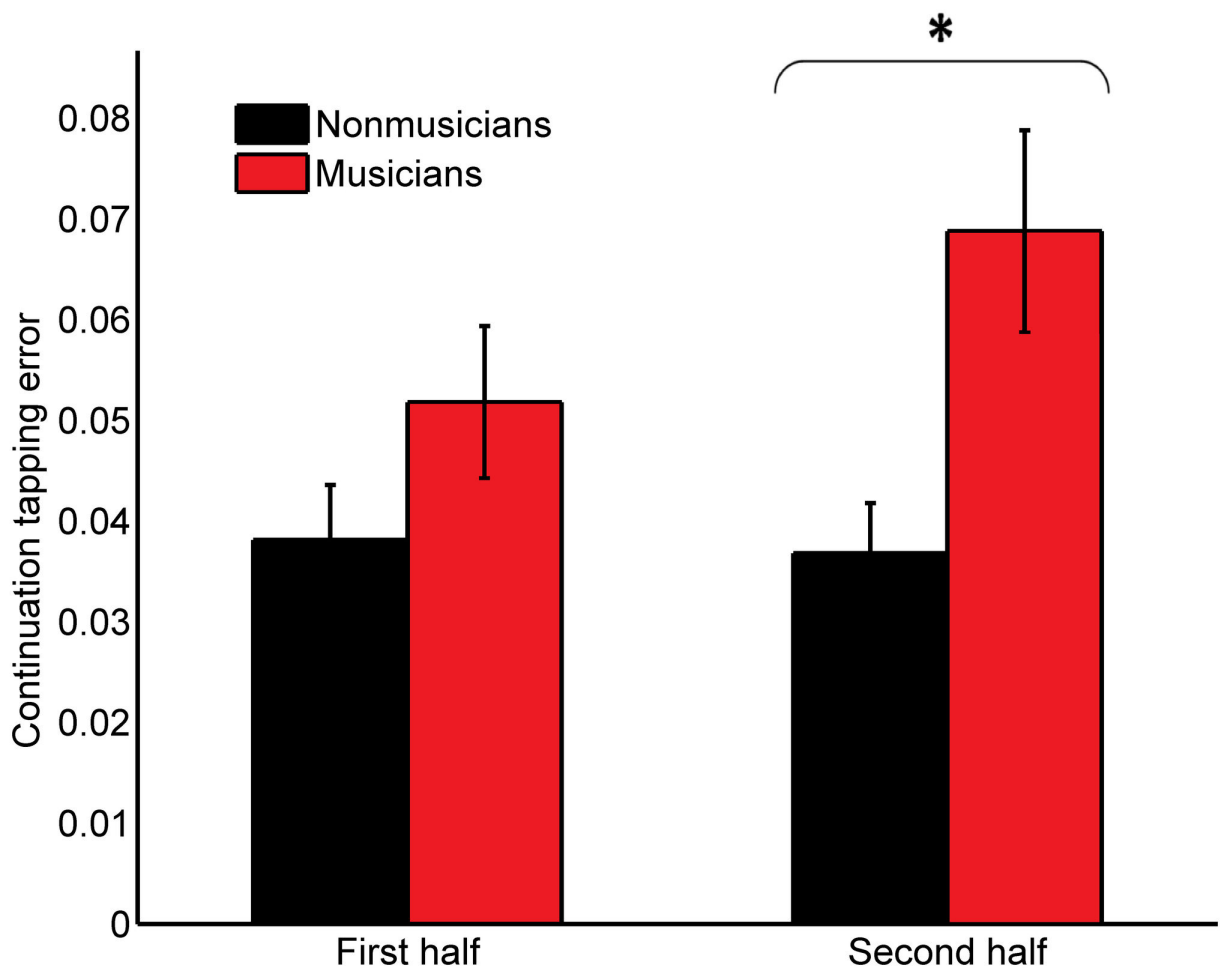
Comparison of musically-trained children (red) and untrained controls (black) showing no group difference in the first half of the continuation tapping period (Wilcoxon rank sum test: p > 0.1) but significantly reduced error in the trained group in the second half (p = 0.0205, zval = -2.32, ranksum = 706). A smaller mean error reflects better ability to keep the beat. (Error bars indicate +/-1SE of the mean.)

**Figure 2:**
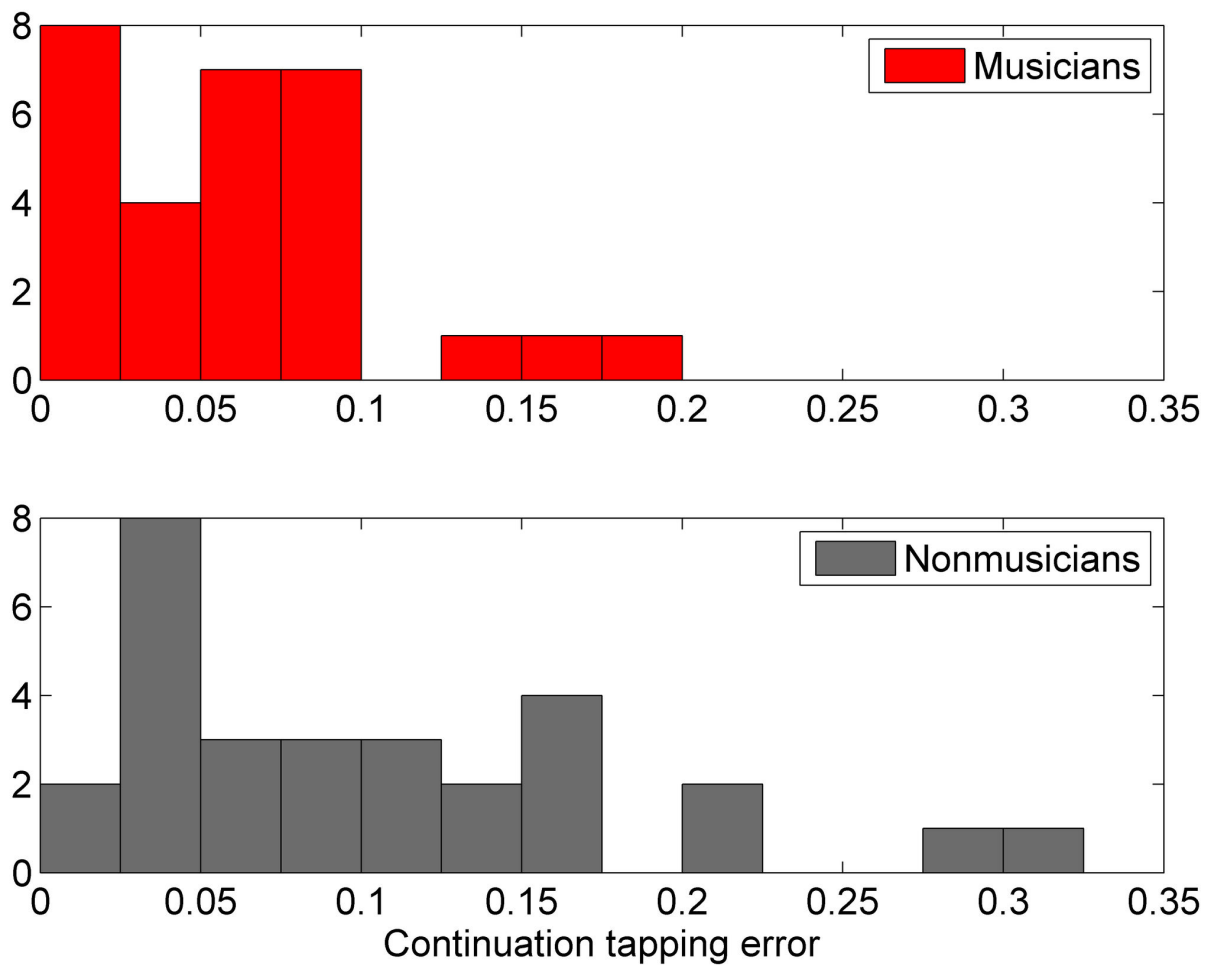
Histograms showing the distribution of tapping accuracy scores in the musically trained and untrained control groups.

To determine whether the enhanced continuation tapping performance in the trained children was due to increased encoding ability or better resilience to memory deterioration, tapping data were divided into first and second halves and analyzed separately. A series of repeated measures ANOVAs were run for each tapping measure, comparing composite performance (averaged across tapping rates) in the first versus the second half as the within-subjects variable and group as the between-subjects variable. For continuation unsigned accuracy, there was a main effect of time (F(1,56) = 4.05, p = 0.0491), a main effect of group (F(1,56) = 5.73, p = 0.0201), and an interaction between time and group (F(1,56) = 5.50, p = 0.0226). Post-hoc Wilcoxon rank sum tests revealed no significant group difference in the first half of the tapping data (p > 0.1). There was, however, a significant group difference in the second half of the tapping data (p = 0.0205, zval = -2.32, ranksum = 706), such that trained children (median error = 3.14%) were more accurate than controls (5.34%) (Figure 1). See Figure 2 for a histogram showing the distribution of unsigned accuracy scores for the two groups. For continuation signed accuracy, there was a main effect of group (F(1,56) = 7.02, p = 0.0105), such that trained children were more accurate than controls, but there was no main effect of time. Finally, for paced variability, there was a main effect of time (F(1,56) = 9.03, p = 0.0040), such that performance in the first half of the test was more variable (median variability 3.95%) than performance in the second half of the test (median variability 5.02%).

## Discussion

We herein assess basic timekeeping abilities in elementary school children using a finger-tapping paradigm. Superior tapping skills have been demonstrated in trained adult musicians [16], but there has been little work to assess the impact of music training on the development of these skills during childhood years. The present findings add to previous work by demonstrating that children who received one year of classroom-based music instruction are significantly more accurate in maintaining a steady beat than their untrained peers. 

Both the trained and untrained groups showed, on average, a negative signed error, meaning that after the pacing stimulus was removed both groups tended to increase the speed of their tapping relative to the target tempo. The trained group, however, sped up to a lesser extent than did the untrained group. The mechanism underlying this increase in tempo is unclear, but it may be related to the tendency for subjects to anticipate the beat when tapping to a pacing stimulus, resulting in a negative asynchrony. Musically-trained individuals show smaller mean negative asynchrony than untrained individuals[19], suggesting that in the general population there is a tendency to overestimate the amount of time that has elapsed since the last beat, and that musical training lessens this tendency. Alternatively, musical training could lessen the tendency to speed up by slowing the preferred rate, as musical training has been linked to slower spontaneous tapping rates[20].

The continuation tapping paradigm used in this study requires that the beat be maintained in memory after the pacing stimuli have stopped, and the greater difference in accuracy between the trained and control groups during the second half of the continuation period suggests that memory may indeed be a factor in how training influences beat-keeping abilities. The initial encoding of the beat period, on the other hand, may be less affected by musical training, as the trained and untrained group performance did not differ during the first half of the continuation period, and synchronization accuracy did not differ in the paced condition, in which the stimulus was present throughout the experiment. Previous research indicates that auditory working memory may also be strengthened by musical training in young children[21], but given the multi-faceted nature of memory it is unknown whether these same capacities underlie any training-related improvement in the ability to maintain a beat over time. 

In both the paced and continuation conditions we found no relationship between training and tapping variability, a measure that has previously been associated with musical experience [22,23]. Our participants, however, had much less musical experience (one year) than participants in prior studies examining synchronization ability and musical experience, which have tested adult professional or semiprofessional musicians. Decreased variability, then, may require either more extensive training or training that is more focused on instrumental performance (in the present study, much of the subjects’ training was dedicated to fostering musicianship skills; participants only began instrumental training after several months of instruction.) On the other hand, the ability to hold a tempo in memory is enhanced after only a year of training that focused in large part on listening skills.

Locking onto temporal patterns in a complex acoustic signal helps the brain to anticipate when meaningful information is likely to occur and streamline allocation of neural resources accordingly, as well as facilitating coordination and planning of associated motor actions. Oscillatory activity may play a role in the dynamic modulation of attention over time[24,25] and in facilitating communication between brain areas such as auditory and motor cortices[26]. Recent research suggests that oscillatory activity associated with cognitive functions such as attention and memory is greater in adult musicians than non-musicians, and that musical training during childhood promotes the development of these underlying oscillatory mechanisms[27] Similarly, musical training may strengthen the mechanisms underlying the brain’s “internal timekeeper,” which is essential to the accurate perception and production of temporal patterns[28]. 

The ability to perceive and predict temporal patterns is also critical to social interaction and communication[29] and there is evidence for some overlap in the neural pathways involved in processing temporal structure in both music and language[30-32], with neural oscillatory activity playing an important role in both rhythm perception and speech processing[33-38]. It is proposed that language difficulties such as dyslexia may result from deficiencies in underlying oscillatory mechanisms and that these deficiencies also result in impaired rhythm perception, which may help to explain the observed relationships between rhythm abilities and reading[9,11-13,39,40]. 

There is evidence to suggest that the perception of meter is malleable with experience, with infants as young as 12 months old demonstrating culture-specific patterns of response to musical rhythms, whereas at 6 months old no cultural specialization is evident[41,42]. Research also indicates that the greater awareness of metrical structure developed through musical training can influence how sequential motor actions are planned and executed, providing a hierarchical framework which facilitates coordination of motor movements over longer timespans[43]. It is possible that one mechanism by which musical training may improve tapping abilities is by providing or reinforcing hierarchical frameworks within which an ongoing beat can more easily be conceived and maintained. Musical activities, particularly ensemble playing, also provide experience in the simultaneous planning and execution of sequential movements over time, which is necessary to maintain a steady beat. 

While the musicianship training provided to the trained group (curriculum is outlined in Table 2) includes tasks relating to rhythm such as call and response rhythmic patterns, these involve hand clapping and have no direct similarities with the tapping measures used in our assessments as far as the equipment (tapping pad) or specific instructions involved. It therefore seems likely that any transfer of ability from the musical training to the finger-tapping task involved generalization of underlying beat perception and beat generation abilities to a new context, rather than direct familiarity with the task per se. It is possible that the children engaged in musical training have developed a greater interest in music-related activities and therefore were more motivated to perform well on a rhythm-related task. However, the fact that the trained and control groups do not differ in tapping variability also suggests that the training effect is not due to a general familiarity or motivation-related effect but is instead the result of a specific enhancement in the ability to maintain a beat after the pacing stimulus is removed.

Our findings contribute to the growing body of research showing selective enhancements in both neural and cognitive function resulting from short-term instrumental music training[27,44-46]. There is significant interest in the role of music education in the development of literacy skills[47-51], and the ability to shape rhythm-related abilities with short-term training is of particular relevance given increasing evidence of links between rhythmic abilities and reading skills[9,11-13].

There is much work still to be done in understanding the mechanisms by which training can strengthen basic temporal processing abilities during critical developmental years, and how this, in turn, may influence emerging literacy skills. Previous work from our lab has demonstrated that even a few years of musical training in childhood can influence the neural processing of sound later in life, years after training has ceased[52], and further research is needed to determine whether the benefits of early musical training for temporal processing are maintained into adulthood. 

## Conclusions

Understanding how classroom music instruction can shape underlying temporal processing abilities has broad educational implications, given converging evidence for the importance of temporal processing for language and literacy skills[9,11-13,53,54]. Our findings indicate that wider benefits of music training can be made accessible to at-risk populations through programs such as Harmony Project, which not only support academic and personal development, but also play a unique and invaluable role in community-building and social cohesion.
