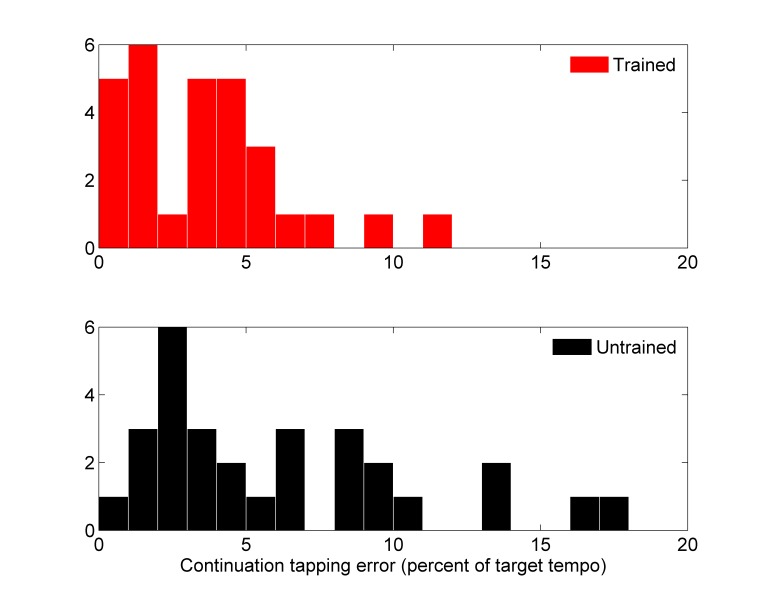# Correction: At-Risk Elementary School Children with One Year of Classroom Music Instruction Are Better at Keeping a Beat

**DOI:** 10.1371/annotation/b6182e63-7d3d-42d7-9624-65c2ce771ae4

**Published:** 2014-01-08

**Authors:** Jessica Slater, Adam Tierney, Nina Kraus

Figure 1 and Figure 2 were old versions of the figures.

Please view the correct version of Figure 1 here: 

**Figure:**
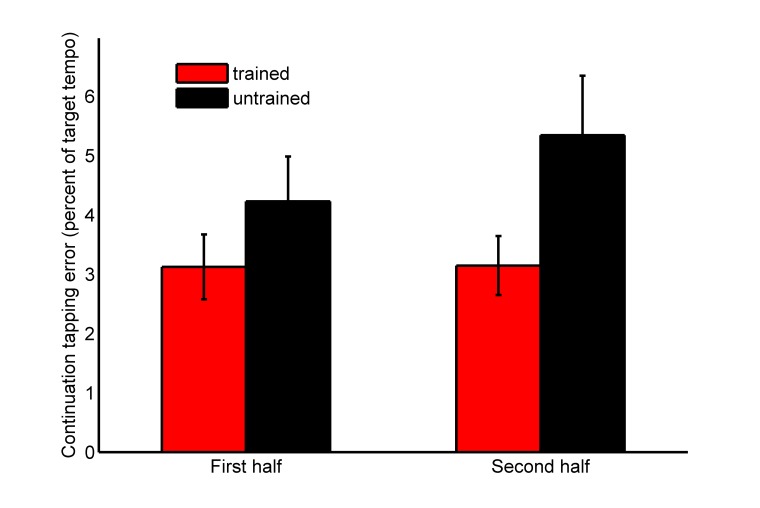


Please view the correct version of Figure 2 here: 

**Figure:**